# Out of pocket and catastrophic health expenditure in Tanzania: recent evidence on the incidence, intensity and distribution

**DOI:** 10.1186/s12913-025-12783-w

**Published:** 2025-05-10

**Authors:** John Massito, Gabriel Hinju

**Affiliations:** 1 Department of Economics, The University of Dodoma, Dodoma, Tanzania; 2 Department of Geography and Economics, University College of Education, University of Dar es salaam, Dar es Salaam, Tanzania; 3 Dar es Salaam, Tanzania

**Keywords:** Out-of-pocket health expenditure, Catastrophic health expenditure, Concentration index, Tanzania

## Abstract

**Background:**

In most low-income countries (LICs), health is mainly financed by out-of-pocket (OOP) expenditures. However, it is claimed that this form of payment causes a massive burden on poor households. This study investigates the catastrophic impact of out-of-pocket health expenditure by estimating the levels, intensities and distribution of catastrophic health expenditure among households in Tanzania.

**Methods:**

The study applied the Wagstaff & va-Doorslaer methodology to measure the incidence and intensity of catastrophic expenditure and the concentration index to measure the distribution of catastrophic expenditure using panel data 2020/2021. Then descriptive-analytical methods such as frequencies, means, and proportions were used to report the results.

**Results:**

The study found that 21.9% (19.1% from rural and 24.6% from urban) of the respondents reported visiting a healthcare facility within four weeks before the survey. Over 50% (53.5% from rural and 57.4% from urban) reported an incidence of illness or injury within the same period. The study also found that among those who utilised health care, about 7.1% (8.4% from rural and 5.7% from urban areas) experienced catastrophic health expenditures. The concentration index (-0.0175 and -0.0638) show that poor households are more likely to experience catastrophic health costs than rich households given the negative values of the indices. This phenomenon is particularly visible in Tanzania, where health insurance is still in its early stages of development.

**Conclusion:**

We conclude that out-of-pocket health expenditures tend to lead to financial catastrophe for poor households, thereby exposing them to more poverty and forcing them to resort to coping mechanisms that compromise their welfare. This necessitates the development of new and reinforced existing systems to protect impoverished households against out-of-pocket and catastrophic healthcare costs.

## Background

In low-income countries (LICs), healthcare financing that relies on out-of-pocket (OOP) expenditures where households directly pay health service providers and inadequate pre-payment system for healthcare has been standard features [13]. This funding model imposes a significant financial strain on impoverished households, often forcing them to bear high healthcare costs that they struggle to cover with limited resources. This situation exacerbates poverty levels as households deplete their savings or incur debt to afford necessary care [12, 22, 29, 40]. The prevalence of poverty in LICs limits the accessibility of prepayment health mechanisms, such as insurance, as a large portion of the population lives below the poverty line and cannot afford insurance premiums [9, 19, 47]. For instance, the 2017/2018 census in Tanzania revealed that 26.4% of the population was poor. During 2013, only 6.6% of the population was covered by the National Health Insurance Fund (NHIF) and about 7.3% by the Community Health Fund (CHF) [38]. OOP expenditures in Tanzania constitute roughly 22% of total health spending, while health insurance accounts for about 8% [23].

When faced with illness, households with limited resources must decide whether to allocate more funds to health care, which might reduce the impact of the illness but also force them to sacrifice other essential expenses or economic activities. Alternatively, they may forego treatment, risking prolonged health deterioration that undermines their ability to earn a living. Opting for treatment can lead to catastrophic financial consequences, pushing households into debt or forcing them to liquidate assets to pay for health care [21, 45]. The saying health is wealth holds economic truth, especially in LICs, where the poor are more susceptible to illness and its related high medical costs, social disruptions, lost income, and suffering [32]. Consequently, substantial OOP payments combined with limited health insurance often result in catastrophic expenditures that need to be considered by policymakers.

Extensive research has explored the factors contributing to catastrophic health expenditures across different countries. Several studies utilising data from Tanzania’s National Household Budget Surveys or primary data indicate that a significant portion of the population experiences catastrophic health spending, with estimates at the 40% threshold of nonfood expenditure revealing incidences from 18% to 26.6% [7, 8, 23, 31, 35, 51]. Kagaigai et al. [23] found that 15% of rural households faced catastrophic expenditures, with a slight variation between members (13%) and non-members (15%) of the improved community health fund. Comparatively, studies in Malawi and Nigeria reported lower incidences of catastrophic health expenditure at 9.3% and 9.6%, respectively [23, 33]. Conversely, higher rates were observed in Kenya and Uganda, at 17.6% and 23%, respectively [12, 32]. Research focusing on the determinants of these expenditures in LICs often examines demographic factors, health status, health-care utilisation, and socioeconomic conditions, highlighting age, education, gender, occupation, income, and chronic illness as significant influences [4, 23, 28, 30, 31, 46].

Further studies, like those by Akinkugbe et al. [2] and Amakom & Ezenekwe [3], investigate health financing and catastrophic payments in Botswana, Lesotho, and Nigeria. Still, their findings are difficult to s Tanzania due to cultural and economic differences. Research on maternal malaria in specific regions also contributes to understanding catastrophic expenditure distribution [5, 33, 39]. Brinda et al. [8] and Kagaiga et al. [23] have closely examined OOP and catastrophic health expenditures in rural Tanzania. However, our study differs by utilising recent panel data from 2020/2021 to capture updated dynamics.

From a policy perspective, it is clear that Tanzania’s current public health insurance schemes, such as the National Health Insurance Fund (NHIF), are insufficient in providing adequate coverage, especially for vulnerable populations and low-income households who continue to face high out-of-pocket (OOP) healthcare costs. In response to these gaps, the government is exploring the introduction of a mandatory health insurance program [25]. This study seeks to empirically examine the relationship between OOP payments, catastrophic health expenditures, and their distribution across the population, offering valuable insights into the challenges and inequities within Tanzania’s healthcare financing landscape. The paper is organised as follows: "Methods" section details the methodology, "Results" section discusses the results, and "Discussion" section offers conclusions and policy recommendations.

## Methods

### Type of data and sources

In establishing the presence of catastrophic health expenditure in Tanzania, the study used the nationally representative panel data of 2020/21 from the National Bureau of Statistics (NBS)-Tanzania to estimate the extent, intensity and variability of the incidence of catastrophic health expenditure. The primary goal of the NPS 2020/21 is to provide high-quality household-level data to the Tanzanian government and other stakeholders for monitoring poverty dynamics and tracking progress on national development initiatives [37]. As an integrated survey covering a variety of socioeconomic indicators, it complements other more narrowly focused survey efforts, such as the Demographic and Health Survey (DHS) on health, the Integrated Labour Force Survey (ILFS) on labour markets, the Household Budget Survey (HBS) on spending, and the National Sample Census of Agriculture (NSCA) (NPS 2020/21 report).

### Sampling in the NPS 2020/2021

The NPS employs a stratified, multi-stage cluster sampling design with four analytical strata: Dar es Salaam, Other Urban Areas on the Mainland, Rural Areas on the Mainland, and Zanzibar. The NPS 2020/21 sample design focused on a subset of homes from the original NPS 2014/15 cohort, known as the Refresh Panel. These families were never included in all three NPS sample designs. The sample we have drawn consists of 3,352 homes from 419 clusters in the NPS 2014/15, which were followed and questioned in the NPS 2020/21. Table 1 shows the distribution of clusters and households across strata in the NPS baseline sample (NPS 2020/21).

**Table 1:** Number of clusters and households in NPS 2020/21 samples as shown in NPS 2014/15 by area

Area	Sampling Design			
	Clusters		Households	
	Expected	Actual	Expected	Actual
Tanzania	420	419	3,360	3,352
*Tanzania mainland*	360	359	2,880	2,872
• Dar es salaam	70	69	560	552
• Other urban	68	68	544	544
• Rural Area	222	222	1,776	1,776
*Tanzania Zanzibar*	60	60	480	480

### Sampling for this study

The sample for this study was extracted from the most recent round of the Tanzania National Panel Survey (NPS) wave 5 of 2020/2021, conducted by the National Bureau of Statistics. For this analysis, households were selected based on the availability of complete data on health-related expenditures and socio-economic indicators. Relevant variables—such as out-of-pocket (OOP) health expenditures, total household consumption, and indicators of catastrophic health spending were constructed using standard definitions from the literature. OOP payments were calculated as the sum of direct health-related expenses reported by households, while catastrophic health expenditure was defined using a threshold of 10% and 40% of total household consumption and non-food expenditure respectively.

### Analytical methods

The analytical methods used in this study are those proposed by Wagstaff and Doorslaer [49] and Xu et al. [53]. The two key variables used in calculating catastrophic health expenditure in this method are total household out-of-pocket (OOP) health expenditure and total household expenditure or total household nonfood expenditure. The household is deemed to have incurred a catastrophic expenditure if its OOP health expenditure as a share of total household expenditure Wagstaff and Doorslaer [49] or total nonfood expenditure Xu et al. [53] surpasses a certain threshold. These methods have also been recommended by the World Health Organization [50] and used in several studies [1, 14, 17, 18, 27, 30, 43, 52, 54] as well as Buigut et al. [10] to investigate similar issues. The details of the methodology are given in the next section.

#### Catastrophic health expenditure

Catastrophic health expenditure (CHE) occurs when medical costs are so substantial that they drive a household’s consumption below the poverty line [26]. There are two main perspectives on defining CHE. The first considers the “capacity to pay” and evaluates health expenditure against the household’s income remaining after meeting basic needs [53]. The second approach assesses the proportion of the total income spent on medical expenses [41]. Regardless of the perspective, a specific threshold is necessary to classify health expenditure as catastrophic.

Various thresholds have been suggested in the literature. Some scholars propose a 40% threshold, where catastrophic expenditure is defined as the ratio of out-of-pocket health expenditures to income left after subsistence basic needs are satisfied, also known as “capacity to pay” by Xu et al. [53]. Other scholars propose a 10% threshold of total expenditure [15, 41, 42, 49].

The selection of a threshold is contentious, as it can seem arbitrary and is influenced by institutional, cultural, and environmental factors. Nonetheless, the significance of the threshold might be less crucial from an analytical standpoint. As Steve Russell [45] points out, a 10% health budget share might not be catastrophic for affluent households, which can reduce luxury spending or access funds through social networks. In contrast, even a 5% share could be catastrophic for poorer households, compelling them to cut back on essential items such as food. Therefore, the financial impact of health expenses varies significantly based on a household’s income level and resource availability.

#### Incidence and Intensity of catastrophic health expenditure

In theoretical terms, the poverty metrics established by Foster, Greer, and Thorbecke in 1984 [20] are frequently employed to evaluate the incidence and intensity of catastrophic health expenditure. This methodology is based on the premise that households incurring substantial health-care costs may significantly deplete their disposable income, reducing their capacity to cover essential needs. To quantify these indices, we calculate the ratio of health-care expenses to either nonfood or total consumption expenditures, as expressed in Eq. 1.

1 $$R_i=\frac{Hexp}{nfexp/Totalexp}\times100$$

In this equation, ${R}_{i}$ represents the fraction of health-care expenditure relative to either nonfood expenditure or total consumption expenditure, $Hexp$ denotes the ’” ’household’s out-of-pocket health expenses, $nfexp$ is the ’” ’household’s average monthly nonfood expenditure, and $Totalexp$ refers to the ’” ’household’s total monthly consumption expenditure. Poverty indices can similarly be adapted to gauge the occurrence and severity of catastrophic expenditures. The initial index calculates the percentage of households whose health care costs exceed a specific proportion of total consumption or nonfood expenditure. This index, known as the headcount, indicates the proportion of households spending beyond a set threshold of their monthly income or expenditure on out-of-pocket health payments [40]. We define a binary indicator $E$ that equals one if ${T}_{i}/{x}_{i}>Z$ (where $x$ represents total household expenditure or capacity to pay, T is the out-of-pocket health-care payments, and $Z$ is the threshold) and zero otherwise. The incidence of catastrophic expenditure is then calculated using Eq. 2:

2 $$H=\frac1N{\textstyle\sum_{i=1}^N}E_i$$

Here, N is the sample size, and H denotes the headcount of households experiencing catastrophic health spending. The headcount index has a notable limitation: it does not convey the severity or depth of the catastrophic expenditure, only the percentage of households affected. To address this, we also compute the catastrophic overshoot to capture the intensity of catastrophic spending. The catastrophic gap, or the average extent by which a household’s health payments exceed the threshold, reflects this intensity. We define the catastrophic gap index ​${O}_{i}$ as follows: ${O}_{i}={E}_{i} (\frac{{T}_{i}}{{x}_{i}}-Z),$ then the catastrophic health payment overshoot is given in Eq. 3.

3 $$O=\frac1N{\textstyle\sum_{i=1}^N}O_i$$

While the headcount H only measures the occurrence of catastrophic expenditures, the overshoot O captures their intensity. The relationship between these two measures is further elucidated by the mean positive overshoot (MPO), calculated as $MPO=O/H$. This index, also known as the mean positive gap (MPG), represents the average amount by which households’ out-of-pocket health expenditures surpass the threshold among those exceeding it, providing a crucial measure of the intensity of catastrophic spending in relation to the headcount H.

### Modelling the distributions of catastrophic health expenditure

One potential disadvantage of the indices discussed in section 2.2.1 is that they do not account for household poverty differences [40]. The headcount, for example, lists all households that have incurred catastrophic health expenditures above the threshold. In contrast, the catastrophic overshoot lists the payments of households whose OOP payments exceed a certain threshold [40]. Distinguishing poor households from non-poor households is an important policy measure issue because, in a real situation, at least a poor household can finance a huge level of OOP health payments by reducing spending on non-basic items like entertainment. However, for a poor household that spends most of its income on necessities, even minimum spending on health can be at the expense of foregone basic needs like food and education. Thus, it is pretty clear that the two groups will differ in the opportunity cost of catastrophic expenditure.

So, to examine the gap in catastrophic health costs between least poor and poor households, the study employed concentration indices, which are extensively used to evaluate inequality in various health-related variables [40, 44]. To assess the disparities, we calculate the headcount and catastrophic overshoot indexes. We borrow a methodology by Chen et al. [11] to calculate these Concentration indexes. The method requires comparing covariance between variables and fractional household ranks based on the ability to pay (ATP). ATP is adjusted for the household’s size and age structure using an equivalency scale [11]. In this study, ATP was measured using ’O’Donnell and Wagstaff’s [40] technique and applied by Chen et al. [11] with per capita household expenditure adjusted for adult equivalency as shown in Eq. 4 below.

4 $$AE={\left(NA+\beta V\right)}^{\mu }$$

NA is the number of adults in the household, and V is the number of children (0–14 years). $\beta$ is the cost of children, and the $\mu$ is the degree of economies of scale. The estimates of the concentration index were obtained using ordinary least squares (OLS) regression of the variables of ATP and the incidence and intensity of CHE on the fractional rank in the ATP distribution, as suggested by O’Donnell and Wagstaff [40] and used by [11].

5 $$2{\sigma }_{\gamma }^{2}\left(\frac{{h}_{i}}{\mu }\right)=\alpha +\beta {\gamma }_{i}+{\varepsilon }_{i}$$

where ${\gamma }_{i}$ is the fractional household rank according to the ATP distribution and ${\sigma }_{\gamma }^{2}$ is the variance. ${h}_{i}$ is the headcount or Overshoot of CHE for household $i,$ and $\mu$ is an estimate of its mean. The OLS estimate of $\beta$ is an estimate of the Concentration index [40]. A positive Concentration index value indicates that poor households have a lower probability of incurring CHE; thus, the distribution here is “pro-poor” [44].

The analysis of catastrophic health expenditure (CHE) is enhanced by considering both weighted and unweighted headcount and overshoot measures, along with their respective concentration indices, denoted as ${E}_{i}({C}_{e})$ and ${O}_{i}({C}_{o})$ respectively. These metrics help to examine how catastrophic expenditures are distributed across different income groups, specifically among poor and non-poor households [40]. The concentration indices typically range from − 1 to 1. A value of 1 signifies that wealthier households predominantly experience catastrophic health expenditures, whereas a value of − 1 indicates that poorer households are more likely to incur such costs [24]. The two indices, i.e. the weighted head count and catastrophic overshoot, are calculated as follows:

6 $$H^w=H^\ast\left(1-C_e\right)\;\mathrm{and}\;O^w=O^\ast\left(1-C_o\right)$$

Where ${H}^{w}$ and ${O}^{w}$ represent the weighted catastrophic headcount and overshoot, respectively. ${H}^{*}$ and ${O}^{*}$ denote the unweighted catastrophic headcount and overshoot. The terms $Ce$ and $Co$ are the concentration indices for the headcount and overshoot, respectively. These formulas adjust the basic headcount and overshoot measures to reflect the concentration of catastrophic expenditure across income groups.

For ethical considerations, assigning greater weight to the excess health payments of poorer households is deemed fair. This approach gives the lowest-income households a weight of two, which decreases linearly with socioeconomic rank, ultimately assigning a weight of zero to the wealthiest households [40]. This weighting scheme ensures that the financial burden on poorer households is given more significance in the overall assessment of catastrophic health expenditure.

### Variable used in the study

Table 2 below describes the variables used in a study. The table shows that there are several variables used in this study ranging from categorical to continuous variables. Categorical variables include expenditure Quintiles, which categorises households according to their consumption expenditure; Household Location, which is a dummy variable that indicates whether the household is located in a rural or urban area; Poverty status, which indicates whether the household is poor or not poor, incidence of illness and insurance status. The other variables are continuous, as shown in Table 2 below.

**Table 2:** Definition of variables used in a study and their measurements

Variable name	Variable definition	Variable Measurement
Household characteristics		
• Household size	The number of people living in the household	continuous
• Incidence of illness	A dummy if illness status(1 = ill, 0 = Otherwise)	Categorical
• Number of people visited the health facilities	The number of people visited health facility	Continuous
• Age	Age of the head of household	Continuous and categorical
• Marital status	A dummy of marital status of the head of household	categorical
• Education	Education level of the head of household	Continuous and categorical
• Insurance	A dummy of insurance status of the household (1 = at least one member has insurance of any form, 0 = Otherwise)	categorical
Expenditure Quintiles	Households are categorised according to their consumption expenditure	Categorical
*Household Location*		
• *Rural/Urban*	The location of the household, rural/urban(1 = rural, 0 = Otherwise)	Dummy
*Poverty status*		
• *Non-poor/Poor*	Dummy for poverty status of the households (1 = Poor, 0 = Otherwise)	Dummy
*Expenditure variables*		
• *Total consumption expenditure*	The household total consumption expenditure per month	Continuous
• *Total health expenditure*	The household total health expenditure per month	Continuous
• *Non-food consumption*	The household non-food consumption per month	Continuous
• *OOP health expenditure*	The household OOP health expenditure per month	Continuous

Source: Author

## Results

### Socioeconomic characteristics of the sampled households

This section presents the descriptive statistics for the sampled households, as detailed in Table 3. In the surveyed sample, approximately 65.9% of rural households and 62.1% of urban households fall within the age range of 15 to 40 years. This demographic distribution aligns with the typical population structure observed in many African countries. The average household size in urban areas is four members. In contrast, in rural areas, there are five members, which is slightly above the average household size of 4.8, as reported in the latest census data for 2020/21.

**Table 3:** Descriptive summary of the population

Household characteristics	Rural	Urban
Household size	5.42	4.30
Incidence of illness	53.5%	57.4%
Number of people visited the health facilities	19.1%	24.6%
Age groups		
• *0–15*	62.1%	46.3%
• *15–40*	65.9%	62.1%
• *40–60*	15.9%	14.9%
• *60* +	5.1%	3.6%
Household Headship		
• *Female*	26.1%	27.9%
• *Male*	74.0%	72.0%
Marital status		
• *Married*	37.1%	56.1%
• *Others*	62.9%	43.9%
*Insurance*		
• *Insurance*	13.9%	14.5%
• *No insurance*	86.1%	85.5%
Education		
• *No schooling*	26.1%	8.8%
• *Primary*	61.7%	49.3%
• *lower Secondary*	10.7%	32.4%
• *upper Secondary*	0.2%	1.5%
• *University*	1.3%	8.0%
Mean consumption expenditure-TZS	96,734.81	173,239.50
Mean Out-Of-Pocket health expenditure-(TZS)	1,900.73	4,668.05
Mean CHE (as a proportion of none food income-TZS)	8.1%	5.2%
Mean CHE (as a proportion of household total income-TZS)	5.1%	6.8%
The proportion of households with health insurance	14.5%	13.9%
Number of observations	2,560	2,149

Source: Authors using NPS 2020/21 data set

The analysis further indicates that about 55% of individuals in rural areas and 57.4% in urban areas reported experiencing illness. Theoretically the urban households have a tendency of perceiving sickness than their rural counterparts, corroborating findings from Moshiro et al. [34]. In addition to that in the context of Tanzania, urban dwellers tend to report more illnesses than their rural counterparts due to a combination of factors including better access to healthcare services, higher health literacy, and more effective health surveillance systems. Urban residents are more likely to recognize symptoms due to higher levels of education and exposure to health information and seek medical attention, leading to higher rates of documented illnesses.

Additionally, urban environments expose people to greater health risks such as air pollution, poor sanitation in informal settlements, and lifestyle-related conditions like obesity, diabetes, and hypertension driven by dietary changes and sedentary behavior. In contrast, rural residents may experience underreporting due to limited healthcare access, reliance on traditional medicine, and lower awareness of health issues. Thus, the higher illness reports in urban areas reflect both actual health burdens and the greater likelihood of illness recognition and documentation. According to Table 3, 19.1% of rural and 24.6% of urban households visited health-care facilities in the four weeks preceding the survey. These figures support the observation that a higher proportion of urban residents report health issues than rural residents.

The analysis reveals that male-headed households are predominant in rural and urban areas, with 74% in rural regions and 72% in urban settings. According to Mukherjee et al. [36], households led by males are generally less likely to experience poverty and tend to influence decisions on resource allocation more significantly. Additionally, the data shows that approximately 15% of surveyed rural households and 14% of urban households have at least one member covered by health insurance.

Regarding economic status, the average monthly per capita consumption expenditure is TZS 96,734.81 (US$ 39.14) in rural areas and TZS 173,239.5 (US$ 70.10) in urban areas. This disparity highlights the typical living conditions in a developing country such as Tanzania. Moreover, the average monthly health-related expenditure for households is approximately TZS 2000 in rural areas and TZS 4668 in urban areas, indicating that health expenses constitute a significant portion of household budgets.

While there is only a slight difference in health insurance coverage between rural and urban households, there is a notable difference in catastrophic health expenditure (CHE). Rural households experience higher CHE when it is measured as a percentage of nonfood income compared to their urban counterparts. However, this difference diminishes when CHE is calculated as a percentage of total household income.

In addition, the table also household characteristics reveal notable differences between rural and urban settings when it comes to marital and educational attainment. In rural areas, a substantial 62.9% of households are married, while only 56.1% in urban areas share this status, indicating a trend toward less traditional marital structures in cities. Education levels illustrate a stark contrast: 26.1% of rural residents have no formal schooling compared to just 8.8% in urban areas, while primary education is more common in rural households (61.7%) than in urban ones (49.3%). Conversely, urban areas show higher percentages of individuals with lower secondary (32.4% vs. 10.7%), upper secondary (1.5% vs. 0.2%), and university education (8.0% vs. 1.3%). The stark contrast in education levels between rural and urban residents can be attributed to factors such as better access to schools and resources in urban areas, economic incentives for higher education, and cultural attitudes toward education. Rural communities often face challenges like transportation issues and limited infrastructure, leading to higher rates of individuals with no formal schooling.

### Categories of the household expenditure quintiles

The calculations of expenditure quintiles were done by dividing the total population into five equal groups based on household monthly real expenditures. The first quintile includes households with the lowest 20% of spending, while the second quintile consists of those whose expenditures fall between the lowest 20% and the 40 th percentile. The middle quintile captures households spending between the 40 th and 60 th percentiles, and the fourth quintile includes those with expenditures ranging from the 60 th to the 80 th percentiles. Finally, the highest quintile encompasses households in the top 20% of expenditures. Each of these quintiles (Table 4) provides a distinct category for analyzing spending patterns across the population.

**Table 4:** OOP and monthly household expenditure across quintiles

Expenditure Quintiles	Rural		Urban	
	Household Monthly OOP (MEAN)	Household Monthly Expenditure (MEAN)	Household Monthly OOP (MEAN)	Household Monthly Expenditure (MEAN)
1. Poorest	861	35,989	1,006	40,283
2.	1,399	64,320	1,922	66,313
3.	1,932	95,616	2,506	97,469
4.	2,585	140,478	3,947	144,034
5. Least Poor	5,733	329,454	8,440	313,799

The expenditure quintiles in Table 4 illustrate significant differences in household spending patterns between rural and urban areas. In the poorest quintile, rural households have an average out-of-pocket (OOP) expense of 861, with total monthly expenditures around 35,989. Urban households in the same category spend slightly more on OOP, averaging 1,006, while their total expenditures reach 40,283. In the second quintile, rural households average 1,399 in OOP costs and 64,320 in total spending, while urban households spend 1,922 on OOP and 66,313 overall. The middle quintile shows rural households incurring 1,932 in OOP expenses, with total expenditures at 95,616, compared to urban households, which spend 2,506 on OOP and 97,469 in total.

In the fourth quintile, rural households average 2,585 in OOP expenses against total spending of 140,478, while urban counterparts spend 3,947 on OOP and 144,034 overall. Finally, in the least poor quintile, rural households report higher total expenditures of 329,454 with OOP costs averaging 5,733, whereas urban households average 8,440 in OOP expenses and 313,799 in total spending. As we move up the quintiles, OOP expenses and total expenditures increase. These trends highlight the varying financial burdens and healthcare costs faced by rural and urban households across different income levels.

### Incidence and intensity of catastrophic health expenditure

We offer assessments of the occurrence and the intensity of catastrophic health expenditure. Initially, we calculate the incidence and severity of these expenditures without weighting, using the 10% and 40% thresholds. Subsequently, we apply these thresholds again to determine the weighted indices, incorporating concentration indices to evaluate whether households with lower or higher incomes are more burdened by catastrophic health expenses. We chose the conventional 10% and 40% thresholds to ensure our findings are comparable with those of other research in the field.

The results from Table 5 reveal that, at the 10% threshold of total consumption expenditure allocated to health care, the proportion of households incurring catastrophic health spending stands at 5.3% in rural areas and 6.6% in urban areas. This statistic signifies that, on average, 5.3% of rural and 6.6% of urban households allocated nearly 10% of their monthly consumption budget to health expenses in the fiscal year 2020/21. For rural households, with an average income of TZS 96,734.62, this translates to an expenditure of TZS 5,127 on health care, while in urban areas, with an average income of TZS 173,239.5, the amount spent on health care is TZS 11,433.8. These figures highlight a significant financial burden, particularly for rural households, who are impoverished and struggle to meet basic subsistence needs. The average overshoot of this threshold-representing the extent by which households exceed the 10% limit-is 0.2% in rural areas and 0.3% in urban areas. The indices calculated at the 40% threshold are derived in the same manner as those at the 10% threshold, allowing for a similar interpretation of the results.

**Table 5:** Catastrophic health expenditure-incidence and intensity at 10% and 40% threshold

	Budget share of OOP, 10% and 40% thresholds			
	*Rural*		*Urban*	
Indices of CHE	*OOP as a share of total expenditure 10%*	*OOP as a share of none food expenditure 40%*	*OOP as a share of total expenditure 10%*	*OOP as a share of none food expenditure 40%*
Catastrophic Head Count (H)	5.3%	8.4%	6.6%	5.7%
SD error	0.053	0.084	0.066	0.057
Catastrophic gape (CG)	0.2%	1.3%	0.3%	0.9%
SD error	0.012	0.054	0.016	0.044
Mean Positive Gap(MPG) = O/H	4.0%	15.8%	5.1%	15.9%

Source: Authors using NPS 2020/21 data set

In contrast to the headcount ratio, which quantifies the number of households experiencing catastrophic health expenses, and the catastrophic gap, which assesses the severity of this spending, the Mean Positive Gap (MPG) provides a measure of how much those who exceed the 10% threshold spend on average. When setting the threshold for catastrophic health expenditure at 40%, households in rural areas that exceed this limit, on average, allocate an additional 15.8% of their total expenditure to health care, culminating in 25.8% of their budget being spent on health. Similarly, those surpassing the 40% threshold in urban areas spend an additional 15.9%, resulting in 25.9% of their total expenditure being directed towards health care. These figures underscore the substantial economic impact on households already struggling with high health-related costs.

### Distributions of the incidence and intensity of catastrophic health expenditure

While the national average Out-of-Pocket (OOP) expenditures for health care provide an overall picture, it fails to reflect the disparities among different households [16, 53]. Specifically, among the large portion of the population without insurance coverage, it is crucial to determine whether wealthier or poorer households bear a greater burden. As discussed in earlier sections, previous measures of catastrophic health expenditure do not differentiate between these economic groups. For instance, the catastrophic headcount counts the number of households incurring catastrophic spending without distinguishing between those who are poor and those who are not. Similarly, the catastrophic gap records the amount by which expenditures surpass a certain threshold but does not account for whether these expenditures are borne by wealthier or poorer households [40].

To address these disparities and provide a more nuanced understanding, we present results for weighted and unweighted indices of catastrophic expenditure and concentration indices. These concentration indices range from − 1 to 1. A positive value indicates that wealthier households are more likely to experience catastrophic health expenditures, whereas a negative value suggests that poorer households are disproportionately affected [24]. Using these indices, we aim to capture the variation in catastrophic health expenditure impacts across different economic strata more accurately.

The CHE incidence and intensity are higher – 5.8% of the total consumption expenditure and 7.9% of the capacity to pay. The CHE incidence and intensity concentration index (Cs) are negative, as shown in Table 6. The statistics in Table 6 also show that the distribution of the catastrophic health expenditure depends on the nature of the denominator variable in calculating catastrophic indices (i.e. whether the fraction of health expenditure is expressed as a percentage of total household consumption expenditure or nonfood expenditure). When we express health expenditure as a proportion of total consumption expenditure, catastrophic payments usually rise with total spending. This tendency explains that the OOP health expenditure budget tends to increase with the total household resources in most low-income countries [48]. Therefore, the weighted indices (Headcount and Overshoot) are larger than the unweighted indices, as shown in Table 6. These values show the percentage of the population experiencing high health expenditures relative to their income. A higher percentage in both weighted and catastrophic headcount indicates a larger proportion of the population facing financial hardship due to health expenses.

**Table 6:** Inequality of catastrophic health expenditure among households based on their poverty status

	Budget share of Out Of Pocket health expenditure	
Catastrophic payment measures	*OOP as a share of total expenditure 10%*	*OOP as a share of non-food expenditure 40%*
*Concentration Index (CH)*	*− 0.0175*	*− 0.0638*
Weighted Headcount (HW)	5.82%	7.96%
Catastrophic headcount (H)	5.72%	7.49%
*Concentration Index C0*	*0.0288*	*− 0.0542*
Weighted Overshoot(OW)	0.25%	1.25%
Catastrophic Overshoot (O)	0.25%	1.19%

Source: Authors using NPS 2020/21 data set

The results of the concentration index indicate that poor households have a higher probability of incurring CHE than rich households, which is evident in developing countries where health insurance is in the embryo stage of development. The Cs of CHE incidence and CHE intensity are all negative, suggesting the existence of pro-poor inequality in CHE in Tanzania, although some CHE intensity Cs were positive. CHE intensity reflected the amount by which households exceeded the CHE threshold. Compared with CHE incidence, CHE intensity measures access to and the use of health care more precisely. Our results imply that CHE incidence and intensity tend to be proportionately distributed in all households, thus indicating that health-care utilisation is strongly associated with household economic level.

## Discussion

In the context of Tanzania, the estimation of catastrophic health expenditure is 5.3% and 6.6% of the total consumption expenditure in rural and urban areas indicates that a significant proportion of households face financial hardships due to health-related expenses. This suggests that a substantial number of Tanzanian families are at risk of falling into poverty or experiencing severe economic strain when seeking healthcare services. Moreover, when looking at the estimates based on nonfood consumption expenditure, which are 8.4% and 5.7% in rural and urban areas, it becomes evident that health expenditure has an even greater impact on rural households than urban households. This could be attributed to various factors, such as limited access to quality healthcare services in rural areas, leading to higher out-of-pocket payments for medical treatment.

These findings underscore the importance of improving healthcare affordability and access in Tanzania, particularly rural areas, to prevent households from facing catastrophic health expenditures. Policy interventions such as expanding social health insurance coverage, increasing government investment in healthcare infrastructure, and promoting health education and prevention programs could help alleviate the financial burden on Tanzanian families and ensure better health outcomes for all citizens.

The negative Concentration Index (Cs) for Catastrophic Health Expenditure (CHE) incidence and intensity indicates that the burden of health-care costs is disproportionately higher among lower-income households. This means that those who can afford it the least bear a larger share of the healthcare costs compared to wealthier households. It highlights the financial hardship vulnerable populations face in Tanzania when accessing healthcare services [6]. The negative Cs value suggests a need for targeted policies and interventions to address the inequitable distribution of healthcare costs and ensure that all individuals, regardless of income level, have access to affordable and quality healthcare. It would be important to consider specific factors contributing to the CHE incidence and intensity concentration among lower-income households in Tanzania for further discussion. This could include the lack of social health protection mechanisms, limited availability of affordable healthcare services, and barriers to accessing healthcare facilities in rural or remote areas.

Addressing these challenges may require expanding health insurance coverage, increasing investment in primary healthcare services, improving healthcare infrastructure in underserved regions, and implementing pro-poor health financing policies [17]. By reducing the financial burden of health-care costs on low-income households, Tanzania can achieve universal health coverage and ensure equitable access to health care for all its citizens.

## Conclusion and policy implication

The analysis of the incidence and severity of catastrophic health expenditure reveals that, on average, about 7.1% of the Tanzanian population faces catastrophic health expenditure each month whenever they seek health care services. The result shows that out-of-pocket health expenditures in Tanzania significantly burden a considerable portion of the population, particularly those residing in rural areas. The high percentage of respondents reporting visits to healthcare facilities (19.1% and 24.6% in rural and urban respectively) within a short period and the incidence of illness or injury (53.5% and 57.4% in rural and urban, respectively) highlights the importance of access to health-care services. Moreover, the finding that a notable proportion (8.4% and 5.7% in rural and urban respectively) of those who accessed healthcare services experienced catastrophic health expenditures indicates a concerning financial impact on households. This suggests that there is a need for strengthening policy interventions to address the issue of affordability and financial protection in health care. The study also has indicated that, in absolute terms, poor households spend slightly more on health care than their counterpart rich households. The high levels of spending on health among the poor, highlight the lack or poor management of relief mechanisms such as health insurance that aim at protecting the poor from catastrophic health expenditures.

In conclusion, the study underscores the urgency of implementing measures to reduce the financial burden of healthcare costs on individuals and families, especially in rural areas. We justify that the effects on affected households, especially poor ones, may not be undermined. Policymakers should consider strategies such as expanding health insurance coverage, increasing access to affordable healthcare services, and strengthening social protection mechanisms to mitigate the catastrophic impact of out-of-pocket health expenditures in Tanzania.

## Limitations of the study

Our analysis is based on the secondary survey data, in which case the accuracy of self-reported health expenditure data may be influenced by recall bias or social desirability bias, where participants may overestimate or underestimate their health spending. Another limitation could be the generalizability of the findings beyond the specific population studied. It’s important to consider these limitations when interpreting the study's findings and to recognise that further research may be needed to confirm and expand upon the results.

## Data Availability

The datasets generated and/or analysed during the current study are available in the National Bureau of Statistics (NBS) repository [https://www.nbs.go.tz].

## Abbreviations

ATP: Ability to Pay
CHE: Catastrophic Health Expenditure
CHF: Community Health Fund
DHS: Demographic and Health Survey
HBS: Household Budget Survey
HW: Weighted Headcount
LIC: Low Income Countries
MPG: Mean Positive Gap
MPO: Mean Positive Overshoot
NBS: National Bureau of Statistics
NHIF: National Health Insurance Fund
NPS: National Panel Survey
NSCA: National Sample Census of Agriculture
OLS: Ordinary Least Squire
OOP: Out-of-pocket
OW: Weighted Overshoot
TNPS: Tanzania National Panel Survey
TZS: Tanzanian Shillings
US$: US Dollar
WHO: World Health Organization
